# Delivery of Antisense Peptide Nucleic Acids to Cells by Conjugation with Small Arginine-Rich Cell-Penetrating Peptide (R/W)9

**DOI:** 10.1371/journal.pone.0104999

**Published:** 2014-08-15

**Authors:** Céline Cordier, Fatima Boutimah, Mathilde Bourdeloux, Florian Dupuy, Elisabeth Met, Patrizia Alberti, François Loll, Gérard Chassaing, Fabienne Burlina, Tula Ester Saison-Behmoaras

**Affiliations:** 1 Muséum National d’Histoire Naturelle, Structure et Instabilité des Génomes, Paris, France; 2 INSERM, U1154, Paris, France; 3 CNRS, UMR 7196, Paris, France; 4 UPMC-Univ Paris 06, Laboratoire des BioMolécules, Université P. et M. Curie, Paris, France; 5 CNRS, UMR 7203, Paris, France; 6 ENS, UMR 7203, Département de Chimie, Ecole Normale Supérieure, Paris, France; University of Helsinki, Finland

## Abstract

Peptide nucleic acids (PNAs) are very attractive antisense and antigene agents, but these molecules are not passively taken into cells. Here, using a functional cell assay and fluorescent-based methods, we investigated cell uptake and antisense activity of a tridecamer PNA that targets the HIV-1 polypurine tract sequence delivered using the arginine-rich (R/W)9 peptide (RRWWRRWRR). At micromolar concentrations, without use of any transfection agents, almost 80% inhibition of the target gene expression was obtained with the conjugate in the presence of the endosomolytic agent chloroquine. We show that chloroquine not only induced escape from endosomes but also enhanced the cellular uptake of the conjugate. Mechanistic studies revealed that (R/W)9-PNA conjugates were internalized via pinocytosis. Replacement of arginines with lysines reduced the uptake of the conjugate by six-fold, resulting in the abolition of intracellular target inhibition. Our results show that the arginines play a crucial role in the conjugate uptake and antisense activity. To determine whether specificity of the interactions of arginines with cell surface proteoglycans result in the internalization, we used flow cytometry to examine uptake of arginine- and lysine-rich conjugates in wild-type CHO-K1 and proteoglycan-deficient A745 cells. The uptake of both conjugates was decreased by four fold in CHO-745 cells; therefore proteoglycans promote internalization of cationic peptides, irrespective of the chemical nature of their positive charges. Our results show that arginine-rich cell-penetrating peptides, especially (R/W)9, are a promising tool for PNA internalization.

## Introduction

Peptide nucleic acids (PNAs) show high sequence specificity in binding to complementary single-stranded RNA and to single- or double-stranded DNA targets. In addition, PNAs have higher chemical stability than natural nucleic acids. Due to these features, PNAs have an exceptional potential for therapeutic applications and diagnostic use [1], [2]. Despite their neutral charge, PNAs do not enter cells more readily than negatively charged oligonucleotides [3]. A variety of methods have been developed to improve PNA uptake into cells, and the currently favored approach involves conjugation to cell-penetrating peptides (CPPs) [4]–[6]. Many groups have attempted to understand what makes a CPP a good PNA carrier, but there are discrepancies in the results most likely resulting from the diversity of the cell systems used, location of target transcript (nuclear or not), types of conjugates (linkers, linker localization, peptides), PNA length, and the methods used to evaluate CPP efficacy (assays for biological function versus fluorescent labels that indicate localization) [7]–[12]. The mechanistic details of how the CPP-cargo conjugates enter the cell remains unclear, although recent data suggest that entry occurs through an energy-dependent endocytic pathway [13]. Cationic CPPs interact with cell glycosaminoglycans, but the mechanisms of cell uptake after cell surface binding appear to be diverse and in some cases are controversial. It is clear that the cell entry mechanism strongly depends on the nature and size of the CPP and also on the type of cargo. Recently, we demonstrated that two steric-blocker tridecamer PNAs that target the HIV-1 poly-purine tract (PPT) sequence induce a sequence-specific and dose-dependent antisense inhibition of luciferase activity in streptolysin-*O* (SLO)-permeabilized HeLa cells engineered to express the luciferase gene with the PPT sequence upstream of the luciferase gene start site [14].

Here, we used the same cell system and fluorescence-based methods to determine the factors involved in the cell uptake efficiency and antisense activity of the anti-PPT PNA H-CCCCCCTTTTCT-Lys (ASPNA) [14] conjugated to the (R/W)9 nonapeptide (H-RRWWRRWRR-NH_2_), which is one of the most efficient among the CPPs tested previously [15], [16]. Recently, it was shown that a PNA containing three Lys residues at the C-terminus and a Cys and a Lys at the N-terminus effectively inhibited activities of miR-155 in cultured B cells [17] and mi2-122 in hepatocarcinoma cells and in human embryonic kidney cells [18]. We have used the same modifications in order to compare intracellular PPT target inhibition by PNA conjugates. In a cell free system, the ASPNA conjugated to (R/W)9 was more active than the Cys-K-ASPNA-K3 conjugate. Therefore, we further investigated (R/W)9-ASPNA uptake and intracellular fate. Neither cell uptake nor antisense activities were strongly affected by whether a disulfide or a maleimide linker was used to conjugate (R/W)9 to the PNA, whereas substitution of the arginine residues in (R/W)9 by lysines led to a six-fold decrease in uptake. In mutant CHO cells in which proteoglycan synthesis is deficient, cell surface binding and internalization of both (R/W)9-PNA and (K/W)9-PNA conjugates were decreased relative to wild-type cells. We then demonstrated that several pinocytic pathways are involved in (R/W)9-PNA internalization in endosomal/lysosomal vesicles. In the presence of the lysosomotropic agent chloroquine (CQ), the (R/W)9-PNA conjugate uptake was comparable to that observed in SLO-permeabilized cells. Treatment with chloroquine not only induced endosome escape of the conjugate but also enhanced cellular uptake.

## Materials and Methods

### PNA oligomers and PNA-CPP conjugates

The H-CCCCCCTTTTCT-Lys anti-PPT PNA (ASPNA), the H-TTTTCCTCTCCCT-Lys scrambled PNA (SCRPNA), PNA-CPP conjugates with or without Texas red (TR), and H-Cys-K-ASPNA- K3 were purchased from Panagene.

### Cell culture

HeLa cells were previously engineered to stably express the firefly luciferase (*Photinus pyralis*) and the *gfp* reporter genes under the control of a bidirectional doxycycline-inducible CMV promoter [14]. The PPT sequence (5′-AAAAGAAAAGGGGGGA-3′) is present upstream of the AUG translation start site of the *luc* gene and the mutated sequence 5′-AAAAGAAGGGGAGGAA-3′ is present upstream of the start site of the *gfp* gene, allowing us to test the sequence-specificity of anti-PPT molecules [14]. These PPT/HeLa cells and the breast adenocarcinoma cell line MCF-7 (American Type Culture Collection) were grown in DMEM medium supplemented with 7% fetal calf serum (FCS). The Jurkat (human T lymphocytes) and the DU145 (human prostate cancer cells) cell lines (American Type Culture Collection) were grown in RPMI medium supplemented with 10% FCS. The human bone osteosarcoma U-2OS cell line (American Type Culture Collection) was cultured in McCoy’s 5A medium, and the Chinese hamster ovary (CHO) cell lines (CHO-K1 and CHO-745, from American Type Culture Collection) were cultured in DMEM F12 medium with 10% FCS. All cell lines were grown at 37°C in a 5% CO_2_ atmosphere. All the media were purchased from Gibco Life Technologies.

### Reversible permeabilization and luciferase assay

SLO (provided by the Institute of Medical Microbiology and Hygiene, Mainz, Germany) was used to reversibly permeabilize PPT/HeLa cells as previously described [14]. Cells (2.6×10^5^ cells/well in 96-well plates) were washed twice and resuspended in Hank’s Balanced Salt Solution (HBSS) with calcium and magnesium (Gibco Life Technologies). Cells were permeabilized at 37°C for 15 min by addition of an optimized amount of SLO (30–60 ng/well) to yield 80–95% permeabilization in the presence of the indicated concentrations of conjugated or unconjugated PNAs. Resealing was achieved by addition of 225 µl DMEM supplemented with 10% FCS, and cells were further incubated at 37°C for 20 min. Cells were then transferred to another 96-well plate (3×10^4^ cells/well) and incubated at 37°C for 1 h. Finally, expression of the *luc* and *gfp* genes was induced by addition of 1 µg/mL doxycycline (Sigma), and cells were cultured for 24 h. Cells were then lysed in Passive Lysis Buffer (Promega), and luciferase activity was quantified with a spectrofluorimeter (Wallac Victor 2 Multi-label Counter, Perkin Elmer) using the Luciferase Assay System (Promega). Background was subtracted from the luminescence readings. Results were normalized to the lysate protein concentration (determined with the Bradford protein assay, Bio-Rad); results are presented as relative light units (RLU/µg protein) and are expressed as the percentage relative to the luminescence obtained in untreated cells. Each data point is the mean of three replicates from each of at least four separate experiments.

### Free uptake of conjugated PNAs

PPT/HeLa cells were seeded (9.5×10^3^ cells/well) in 96-well plates and grown overnight. Cells were washed with PBS (Gibco Life Technologies) and then incubated with PNAs or peptide-PNA conjugates in the presence or absence of 150 µM chloroquine (Sigma) in 50 µL of serum-free Opti-MEM medium or in DMEM (Gibco Life Technologies) with 7% FCS for 4 h. Luciferase expression was induced by addition of 150 µL doxycycline (1 µg/mL) in DMEM medium, and then cells were cultured for another 24 h. For cell viability assays, cells were treated in the same way, and viability was evaluated with the CellTiter 96® Aqueous Non-Radioactive Cell Proliferation Assay (Promega) according to the manufacturer’s instructions.

### Fluorescence microscopy of live cells

In all experiments, 8-well, chambered cover glass microslides (IBIDI, Biovalley) were used. Cells (4×10^4^ cells/well) were seeded in the chambers and cultured overnight at 37°C. Cells were washed with HBSS (Gibco Life Technologies) and incubated with the indicated concentrations of fluorescent conjugate or PNA in 150 µL Opti-MEM at 37°C for indicated times. Culture medium was replaced with DMEM without phenol red (Gibco Life Technologies) for microscopic observation of live cells using two inverted microscopes: The first was a Leica microscope DMIRE2 equipped for epi-illumination with an Osram HXP R 120W 45C lamp and Rooper Coolsnap CCD camera. Acquisitions were made with the Metamorph software using a 100x oil immersion objective (numerical aperture of 1.4) and a CY3 filter (EX BP 545/30, EM BP 610/75). The second microscope was a Zeiss Axio Observer.Z1 with a structured illumination system (ApoTome). Images were taken with an OrcaR2 (Hamamatsu) camera using a 63x oil immersion objective (numerical aperture of 1.4) and a 43 DsRed shift free filter (EX BP 550/25, EM BP 605/70). Acquisitions were made with the AxioVision4.8 software. Images were analyzed using the Image J software.

### Fluorescence-assisted cell sorting (FACS) analysis

For uptake kinetics, exponentially growing PPT/HeLa cells were plated in 48-well plates (4.0×10^4^ cells/well). The next day, cells were washed with PBS and incubated with TR-labeled conjugate or naked PNAs, as described above. After incubation, cells were trypsinized (Gibco Life Technologies) and washed twice with PBS. Fluorescence analysis was performed with a C6 flow cytometer (Accuri) using the Cy5 channel (FL3). The background-free mean fluorescence values were calculated by subtracting the mean fluorescence of untreated cells from the obtained mean fluorescence. The error bars shown represent the standard square deviation of at least three independent experiments. Based on cell size (FSC) and granularity (SSC), only the live cell fraction was analyzed.

### Uptake experiments

#### Energy depletion

Cells were incubated with the PNA conjugates in the presence of 10 mM sodium azide and 6 mM deoxy-D-glucose at 37°C or at 4°C for 1 h. Medium was discarded and replaced with DMEM without phenol red. Cells were observed with a Zeiss Axio Observer.Z1 microscope or collected for FACS analysis.

#### Heparin competition

Cells (4×10^4^ cells/well), seeded 17 h earlier, were pre-treated with 5 µg/mL heparin sodium salt from porcine intestinal mucosa (Sigma) in Opti-MEM medium for 30 min. Cells were then incubated at 37°C with 1 µM conjugate in the presence of 5 µg/ml heparin for 1 h and were washed with PBS before FACS analysis.

#### Chloroquine treatment

Cells in IBIDI microslides were incubated with 150 µM CQ and 1 µM TR-labeled conjugates for 4 h. Cells were then directly analyzed by FACS and microscopy.

#### Co-localization experiments

Cells in Ibidi microslides, seeded 17 h earlier at 4×10^4^ cells/slide, were incubated with 1.5 µM (R/W)9-PNA-TR conjugate with 25 µg/ml Alexa 488-transferrin, 0.25 mg/mL Alexa 488-dextran, or 10 µg/mL Alexa 488-cholera toxin subunit B (Life Technologies) in Opti-MEM medium at 37°C for 2 h. Control cells were not treated with PNA conjugate. Medium was replaced with DMEM without phenol red for image acquisition with a Zeiss Axio Observer.Z1 microscope. For lysosome labeling, cells were treated for 4 h with 1.5 µM (R/W)9-ASPNA-TR conjugate and then cultured for 18 h in conjugate-free medium. At 2 h before image acquisition, 1 µM LysoTracker Green DND-26 (Life Technologies) was added. We used Icy imaging software, (http://icy.bioimageanalysis.org/) [19] to quantify our data. We used the “Protocols editor” plugin to run a specific protocol to quantify co-localisation spots (http://icy.bioimageanalysis.org/protocol/Colocalizer).

### Statistical analyses

All the statistical analyses were performed using the Mann-Whitney test with the SOFA Statistics software. Errors bars represent the standard deviation of at least three independent experiments. *: *P*<0.05; **: *P*<0.01; **: *P*<0.001.

## Results

### (R/W)9-ASPNA conjugate efficiently inhibits intracellular luciferase activity

**Figure 1:**
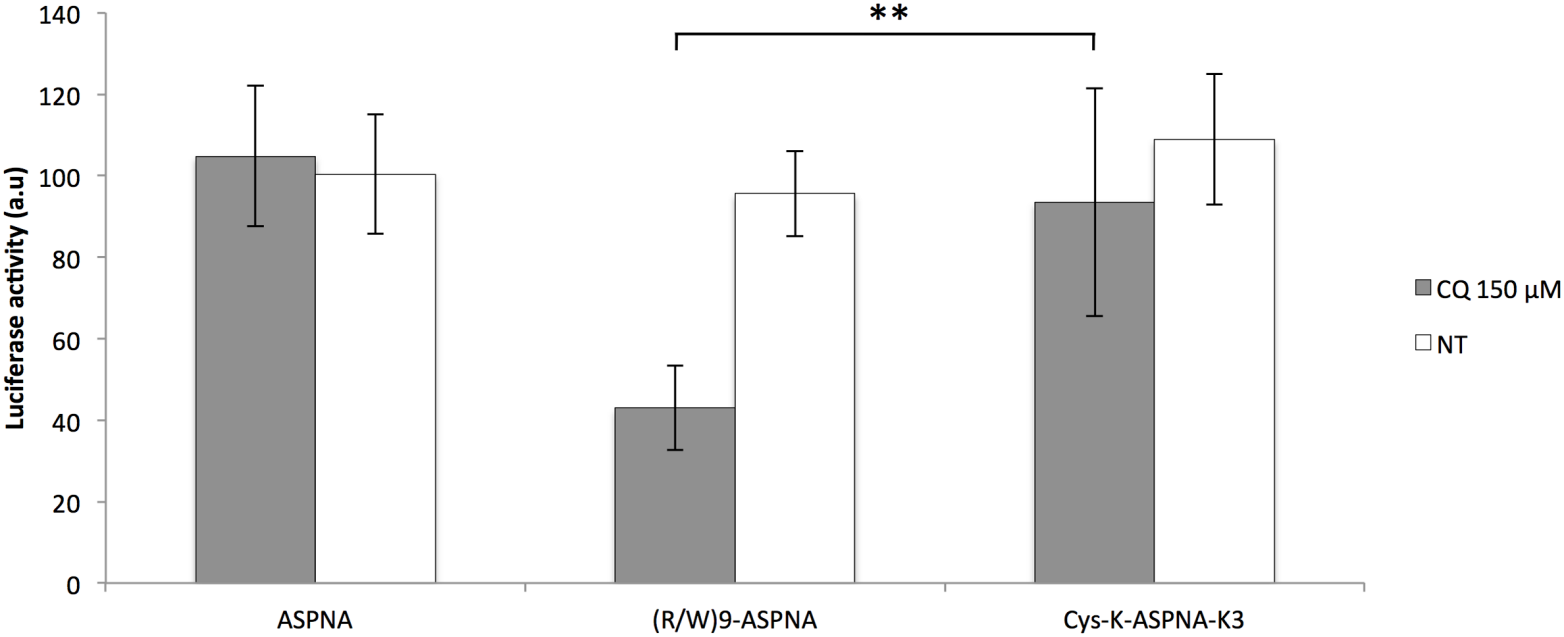
Inhibition of luciferase expression by ASPNA conjugated to peptides. (A) Relative luciferase activity in PPT/HeLa cells incubated with conjugated or unconjugated ASPNA as indicated at 1.5 µM concentrations with (grey bars) or without (white bars) 150 µM chloroquine (CQ) for 4 h in serum-free Opti-MEM medium. Luciferase activity was measured 24 h after addition of doxycycline and expressed as luminescence/mg of protein. Percentages are relative to luciferase activity in untreated cells. Values are the mean ± SD of at least three independent experiments.

We first evaluated the antisense activity of unconjugated PNA (ASPNA) and (R/W)9-ASPNA and Cys-K-ASPNA-K3 conjugates (**see** **Table 1** **for sequences**) in PPT/HeLa cells in the presence of chloroquine (CQ). These cells express the *luciferase* gene with an upstream PPT sequence that is under the control of the doxycycline-inducible CMV promoter. As expected, the unconjugated PNA was not active in cells, whereas the (R/W)9-ASPNA conjugate inhibited luciferase activity by 60% relative to untreated control cells (**Fig. 1**). The Cys-K-ASPNA-K3 conjugate was much less active under these conditions than the (R/W)9-PNA conjugate. It is noteworthy that this effect was obtained only when cells were co-incubated for 4 hours with 1.5 µM conjugate and 150 µM CQ; CQ is a lysosomotropic agent that accumulates in endosomes and lysosomes and enhances the cytosolic delivery of cargoes attached to CPPs [20].

**Table 1:** Unconjugated and conjugated PNA molecules used in this study.

ASPNA	H-TCCCCCCTTTTCT-Lys
SCRPNA	H-TTTTCCTCTCCCT-Lys
(R/W)9	RRWWRRWRR-NH_2_
(K/W)9	KKWWKKWKK-NH_2_
	H-Cys-K-ASPNA-K3
H-Cys-PNA- (Lys, TR)	
S (mal)	
S	
H-Cys-(R/W)9 or (K/W)9	

PNA and peptide sequences are written from the N to the C terminus. PNA, peptide nucleic acid; S-S, disulfide linker; mal, maleimide; TR, Texas red.

**Figure 2:**
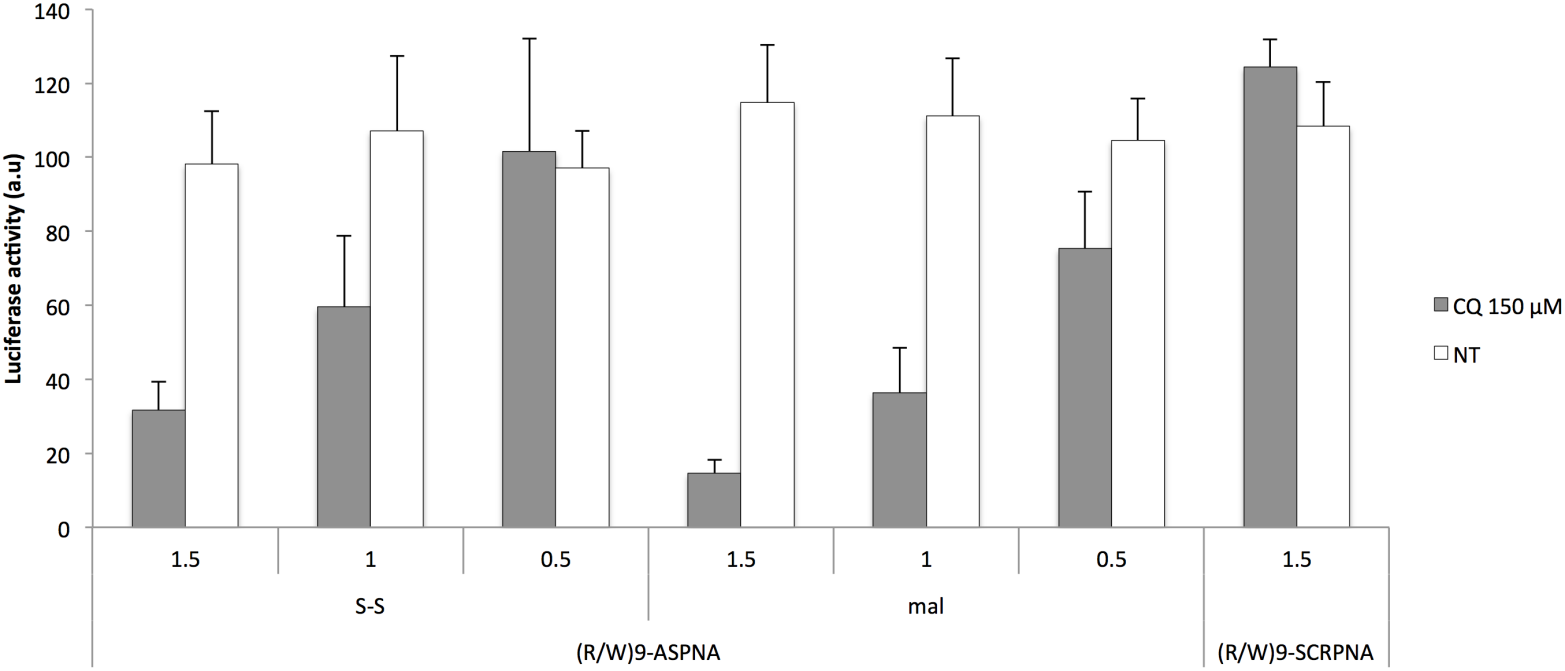
Inhibition of luciferase expression by (R/W)9-ASPNA. Comparison of disulfide or maleimide linker containing (R/W)9-ASPNA in dose response. Relative luciferase activity in PPT/HeLa cells incubated with conjugated ASPNAs with (grey bars) or without (white bars) 150 µM chloroquine (CQ) for 4 h in serum-free Opti-MEM medium. Luciferase activity was measured 24 h after addition of doxycycline and is expressed as luminescence/mg of protein. Percentages are relative to luciferase activity in untreated cells. Values are the mean ± SD of at least three independent experiments. ASPNA, antisense PNA; SCRPNA, scramble PNA; S-S, disulfide linker; mal, maleimide linker.

To determine whether the conjugation of the nonapeptide affects the antisense activity of the PNA, the conjugated and unconjugated PNAs were introduced into SLO-permeabilized cells and luciferase activity was measured after 24 hours, as previously described [14]. Unconjugated and (R/W)9-conjugated ASPNAs both inhibited luciferase activity by about 70% at 1 µM concentration (**Fig. S1**), indicating that unconjugated and (R/W)9-conjugated ASPNAs have the same ability to recognize their intracellular mRNA target and reduce gene expression. The antisense effect was target specific because a scrambled sequence (SCRPNA) did not affect luciferase activity (**Fig. S1**). The nature of the linkage between the CPP and PNA did not strongly affect the conjugate efficiency. In the presence of CQ, both the disulfide conjugate and the maleimide conjugates showed strong dose-dependent and sequence-specific inhibition of luciferase activity (**Fig. 2**); the maleimide-linked (R/W)9-PNA conjugate was slightly more inhibitory (85% ±4 at 1.5 µM) than the disulfide bond-linked conjugate (68% ±8 at 1.5 µM). Cell viability was >85% for all PNA conjugates in the presence of CQ (**Fig. S2**). These results clearly show that the presence of a cleavable disulfide bond between (R/W)9 and PNA is not essential for cell uptake, whereas the release from endosomal- and membrane-bound compartments is crucial for the antisense activity. Moreover, serum proteins did not affect the internalization or biological activity of either conjugate. (R/W)9-ASPNA conjugates elicited similar antisense activities when added to cells in serum-free medium or medium containing 7% FCS (**Fig. S3**).

### (R/W)9-PNA conjugates are internalized via pinocytosis

#### Kinetics and energy-dependence of (R/W)9-PNA-TR internalization

Internalization of extracellular material can occur through different mechanisms, including phagocytosis or pinocytosis. Pinocytosis can be categorized as macropinocytosis or clathrin-dependent (CDE) or clathrin-independent (CIE) endocytosis. To study how the (R/W)9 peptide delivers ASPNA into cells, we labeled the disulfide-linked (R/W)9-ASPNA conjugate with Texas red [(R/W)9-ASPNA-TR]. The fluorescent probe was attached to the C-terminus of PNA because the (R/W)9 peptide is less stable than PNA and may be detached upon disulfide bridge reduction [21]. We made all our observations in live cells as fixation procedures can cause major artifacts and modify the intracellular distribution [22]. We did not use chemical inhibitors for specific endocytosis pathways because recent studies showed that they lack selectivity and may give misleading results [23].

**Figure 3:**
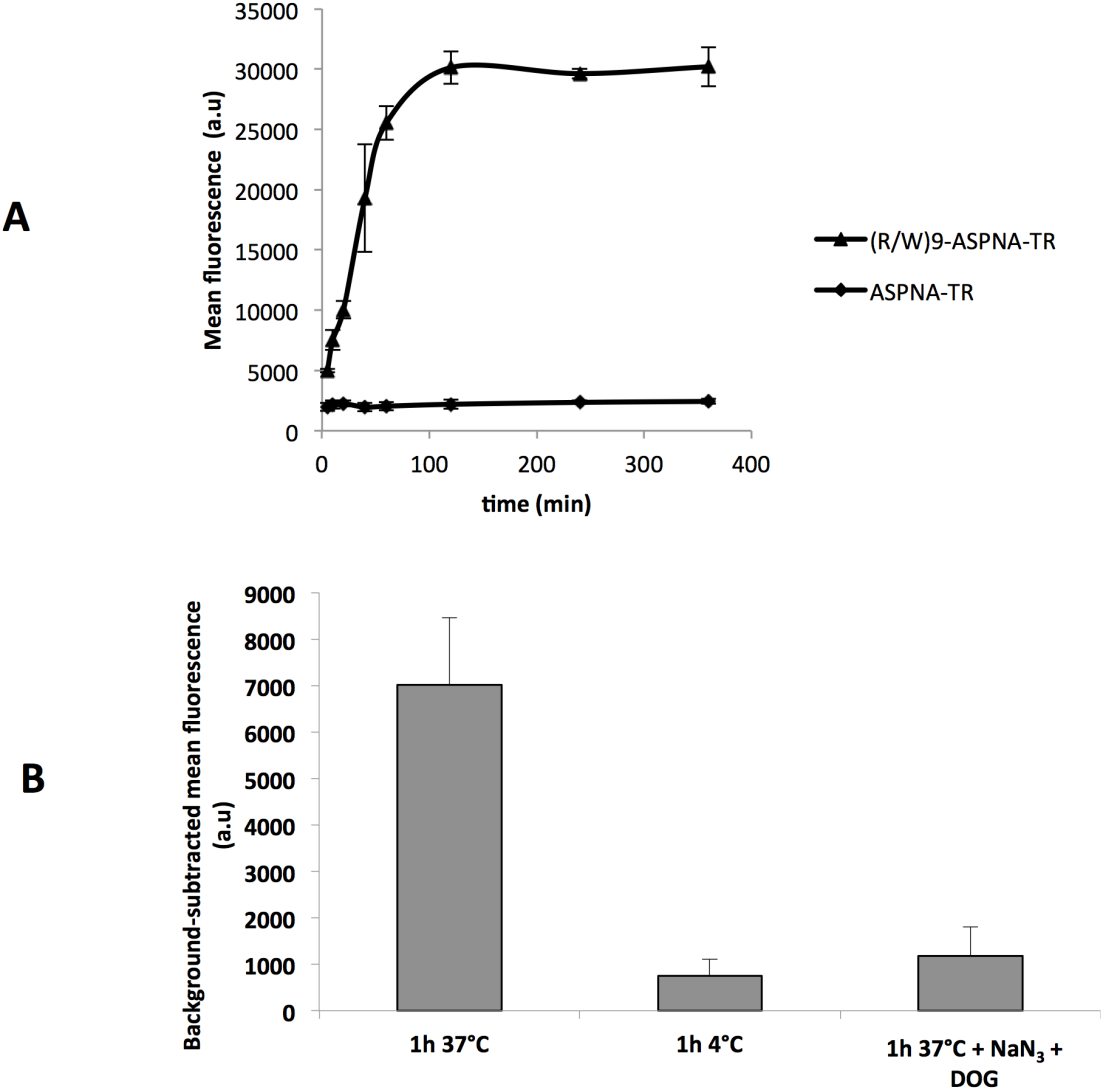
Kinetics and energy-dependence of (R/W)9-ASPNA-TR uptake into PPT/HeLa cells. (A) Cells were incubated with 1 µM (R/W)9-ASPNA-TR or with 1 µM ASPNA-TR for the indicated times. Fluorescence was measured by FACS, and data shown are the mean ± SD of three independent experiments. (B) PPT/HeLa cells were incubated with 1 µM (R/W)9-ASPNA-TR at 37°C or at 4°C or with 1 µM (R/W)9-ASPNA-TR and 10 mM sodium azide (NaN_3_) and 6 mM deoxyglucose (DOG) to deplete cellular ATP at 37°C for 60 min. The histogram shows the fluorescence values (means ± SD of four independent experiments) measured by FACS analysis.

First, we determined the kinetics of (R/W)9-ASPNA-TR uptake by measuring the cell-associated fluorescence by FACS after trypsin digestion. Analysis of the FACS profiles and of the mean cell fluorescence values clearly indicated a progressive uptake over 60 min after which levels reached a plateau that persisted for at least 6 hours (**Fig. 3A**). No uptake was observed with unconjugated ASPNA-TR. We then examined (R/W)9-ASPNA-TR uptake under conditions of energy depletion by incubating cells at 4°C or in the presence of the energy-depleting mixture of deoxyglucose (DOG) and sodium azide (NaN_3_) for 1 hour. Quantification of internalized conjugates by FACS analysis confirmed that (R/W)9-ASPNA-TR uptake was energy dependent (**Fig. 3B**).

#### CQ enhances conjugate cellular uptake and release from endosomes

**Figure 4:**
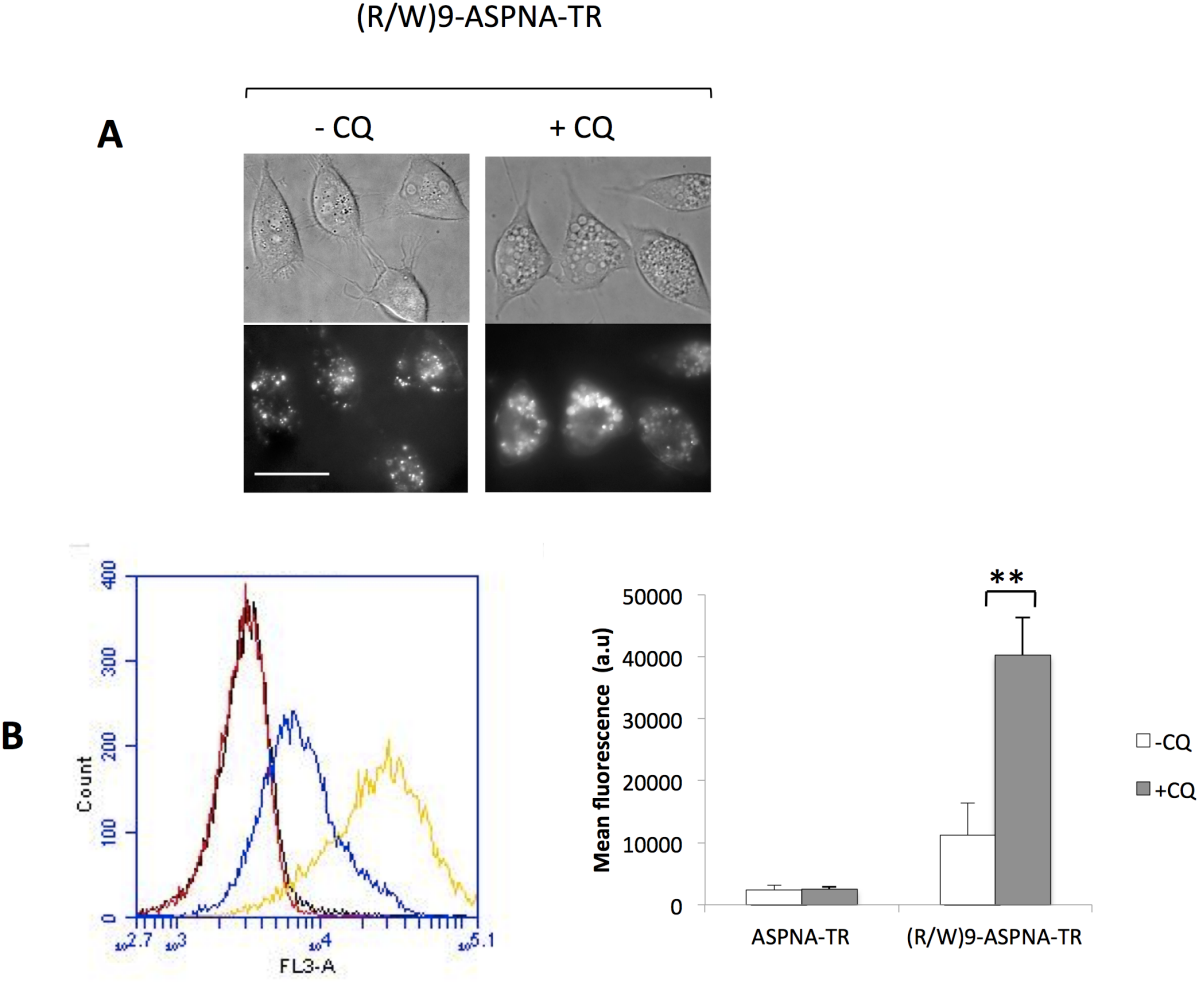
Effects of the chloroquine on (R/W)9-ASPNA internalization. (A) PPT/HeLa cells were incubated with 1 µM (R/W)9-ASPNA-TR with (+CQ) or without chloroquine (–CQ) for 4 h prior to microscopic observation. Images are the projection of all z-stacked signals (0.2 µm between each image). Light field (upper panels) and fluorescent images (lower panels) are shown. Scale bar: 30 µm. (B) Representative FACS data of cell uptake (TR fluorescence) of ASPNA-TR in the presence of CQ (red curve) or in the absence of CQ (black curve) and of (R/W)9-ASPNA-TR in the presence of CQ (yellow curve) or in the absence of CQ (blue curve). Fluorescence quantification (mean ± SD; n = 5) is shown in the histogram.

In the absence of CQ, the (R/W)9-ASPNA-TR conjugate showed a punctuate distribution in the cytosol with rather small puncta that were well distributed, typical of endosomal sequestration (**Fig. 4A**). Upon addition of 150 µM CQ, the intensity of the TR signal inside the vesicles and the vesicle size increased (**Fig. 4A**). Quantification by FACS analysis clearly showed that (R/W)9-ASPNA-TR uptake was four times higher in the presence of CQ than in its absence (**Fig. 4B**). Since there was no modification of the fluorescence emission spectra of the conjugate in the presence of CQ (data not shown), the enhancement of fluorescence was attributed to CQ-induced increase of conjugate uptake. A similar CQ-induced uptake enhancement has been reported for DNA transfected in complex with polycationic polymer polyethylenimine [24].

#### Pinocytotic pathways are involved in (R/W)9-ASPNA-TR internalization

**Figure 5:**
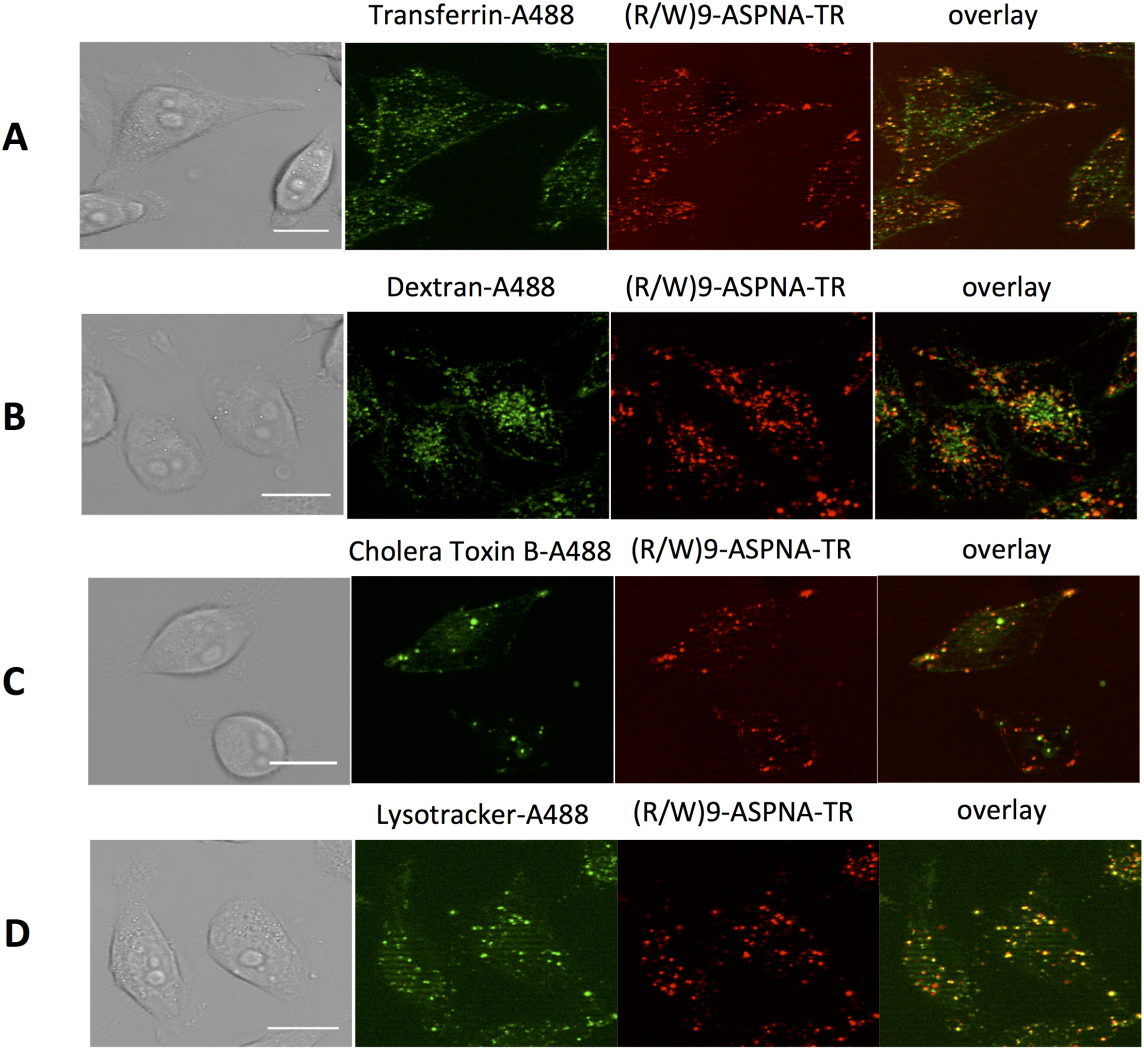
Endocytosis pathways involved in (R/W)9-ASPNA-TR cell uptake. PPT/HeLa cells were incubated with Opti-MEM medium containing 1.5 µM (R/W)9-ASPNA-TR and (A) 25 µg/ml Alexa Fluor 488-transferrin, (B) 0.15 µM Alexa Fluor 488-dextran or (C) 10 µg/ml Alexa Fluor 488-cholera toxin subunit B at 37°C for 2 h and then analyzed. (D) Cells were incubated with 1.5 µM (R/W)9-ASPNA-TR conjugate for 4 h and grown for 18 h in conjugate-free medium. Lysotracker Green DND-26 was then added for 2 h and cells were observed using a Zeiss Axio Observer.Z1 microscope. Left panels show differential interference contrast images, middle panels fluorescence images, and right panels show overlays of the fluorescent images (i.e., merged green and red channels). Scale bar: 20 µm. Extents of co-localisation of (R/W)9-ASPNA-TR conjugate with the following were determined using the Icy imaging software (http://icy.bioimageanalysis.org/): Alexa Fluor 488-transferrin, 45.22±6.92%; dextran, 42±0.29%; cholera toxin, 47.91±0.01%; and Lysotracker Green DND-26, 48.85±5.09%.

CDE is the best characterized endocytic pathway and many macromolecules are specifically taken up in clathrin-coated vesicles [25]. To test whether CDE is involved in (R/W)9-ASPNA-TR internalization, we incubated PPT/HeLa cells with (R/W)9-ASPNA-TR in the presence of Alexa Fluor 488-labeled human transferrin (hTF), which is considered to be a good CDE marker because in its iron-bound form it binds to the transferrin receptor and is taken up via CDE [26]. Fluorescence microscopy analysis showed that hTF and (R/W)9-ASPNA-TR were localized in the same compartments (**Fig. 5A**). Kinetic studies indicated that after 60 min a fraction of labeled conjugate was co-localized with transferrin; after 2 hours 45.22±6.92% of the conjugate was co-localized with transferrin. Macropinocytosis is a non-selective endocytic mechanism of internalization of suspended macromolecules, particularly fluid-phase markers, such as high-molecular-weight dextran. We incubated PPT/HeLa cells with (R/W)9-ASPNA-TR in the presence of Alexa Fluor 488-labeled dextran and observed 42.91±0.01% co-localization with (R/W)9-ASPNA-TR (**Fig. 5B**). The active fraction of cholera toxin is internalized by CIE from lipid rafts, which are cell membrane micro-domains enriched in cholesterol and sphingolipids [27]. **Figure 5C** shows that (R/W)9-ASPNA-TR co-localized with the Alexa Fluor 488-labeled B subunit of the cholera toxin (47.91±0.01%).

PPT/HeLa cells were co-incubated with (R/W)9-ASPNA-TR and Alexa 488-labeled LysoTracker green, a specific lysosomal dye. Co-localization analysis of all the studied vesicles revealed an overlapping of 48.85±5.09% of (R/W)9-ASPNA-TR-positive and LysoTracker-positive structures, indicating that at least part of the conjugate is targeted to lysosomes through the endo-lysosomal pathway (**Fig. 5D**). Together, these results show that macropinocytosis, CDE, and caveola-mediated endocytosis all contribute to (R/W)9-ASPNA-TR uptake and transport into lysosomes.

### Replacement of arginines by lysines in CPP decreases cell uptake and antisense activity of ASPNA conjugates

**Figure 6:**
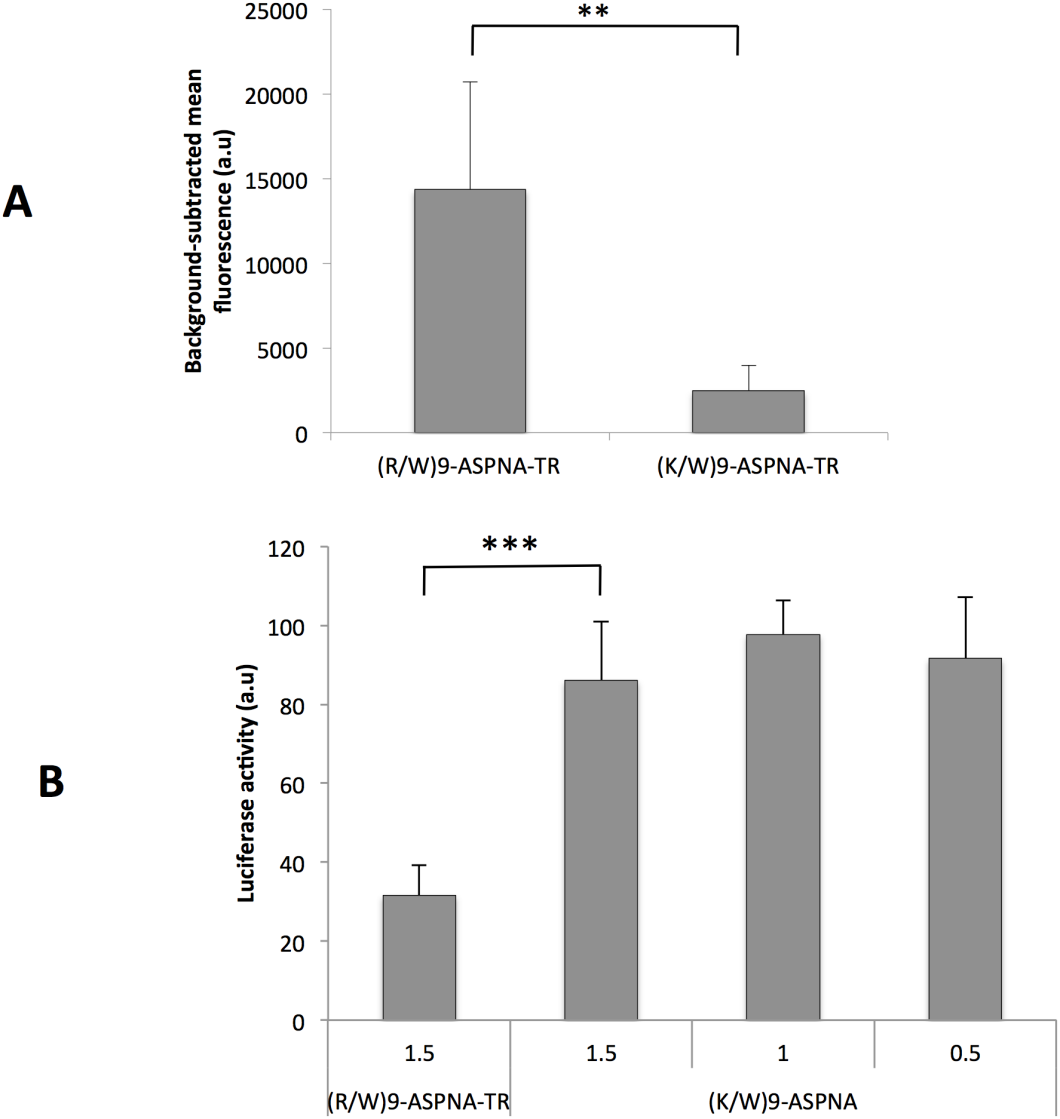
Effect on the substitution of (R/W)9 arginines by lysines on the uptake and antisense activity of the conjugated ASPNA. (A) PPT/HeLa cells were incubated with 1 µM (R/W)9-ASPNA-TR or 1 µM (K/W)9-ASPNA-TR conjugates for 4 h. Intracellular fluorescence was measured by FACS, and the background fluorescence of the cells incubated with ASPNA-TR was subtracted. Histograms show the quantitative determinations (means ± SD; n = 6). (B) PPT/HeLa cells were incubated with the indicated concentrations of (R/W)9-ASPNA-TR or (K/W)9-ASPNA-TR in serum-free Opti-MEM medium and 150 µM chloroquine for 4 h. Luciferase expression was induced by addition of 150 µL doxycycline. After 24 h cells were lysed and luciferase activity was measured. Values (means ± SD of at least seven independent experiments) were normalized to the average value of cells not treated with PNA.

To explore how arginine and lysine residues contribute to cell surface binding and cell uptake of CPP-PNA conjugates, we compared the activity of (R/W)9-ASPNA-TR to that of (K/W)9-ASPNA-TR, in which all the arginine residues of (R/W)9 were substituted by lysines. We incubated PPT/HeLa cells with 1 µM (R/W)9-ASPNA-TR or 1 µM (K/W)9-ASPNA-TR and quantified cell-bound TR fluorescence of live cells. Uptake of (K/W)9-ASPNA-TR was six-fold lower than that of (R/W)9-ASPNA-TR (**Fig. 6A**), indicating that arginines in the CPP were critical for cell uptake. Similar results were obtained in four different human cell lines (**Fig. S4**). Consistent with this observation, (K/W)9-ASPNA-TR did not have any effect on luciferase activity under conditions where (R/W)9-ASPNA induced 70% inhibition (**Fig. 6B**). It should be emphasized that both conjugates induced equivalent inhibition of luciferase activity in SLO-permeabilized cells (**Fig. S5**). Therefore, the lack of intracellular activity was not due to differences in the affinity for the target, but only to differences in cell uptake.

### (R/W)9-ASPNA-TR endocytosis is glycosaminoglycan dependent

**Figure 7:**
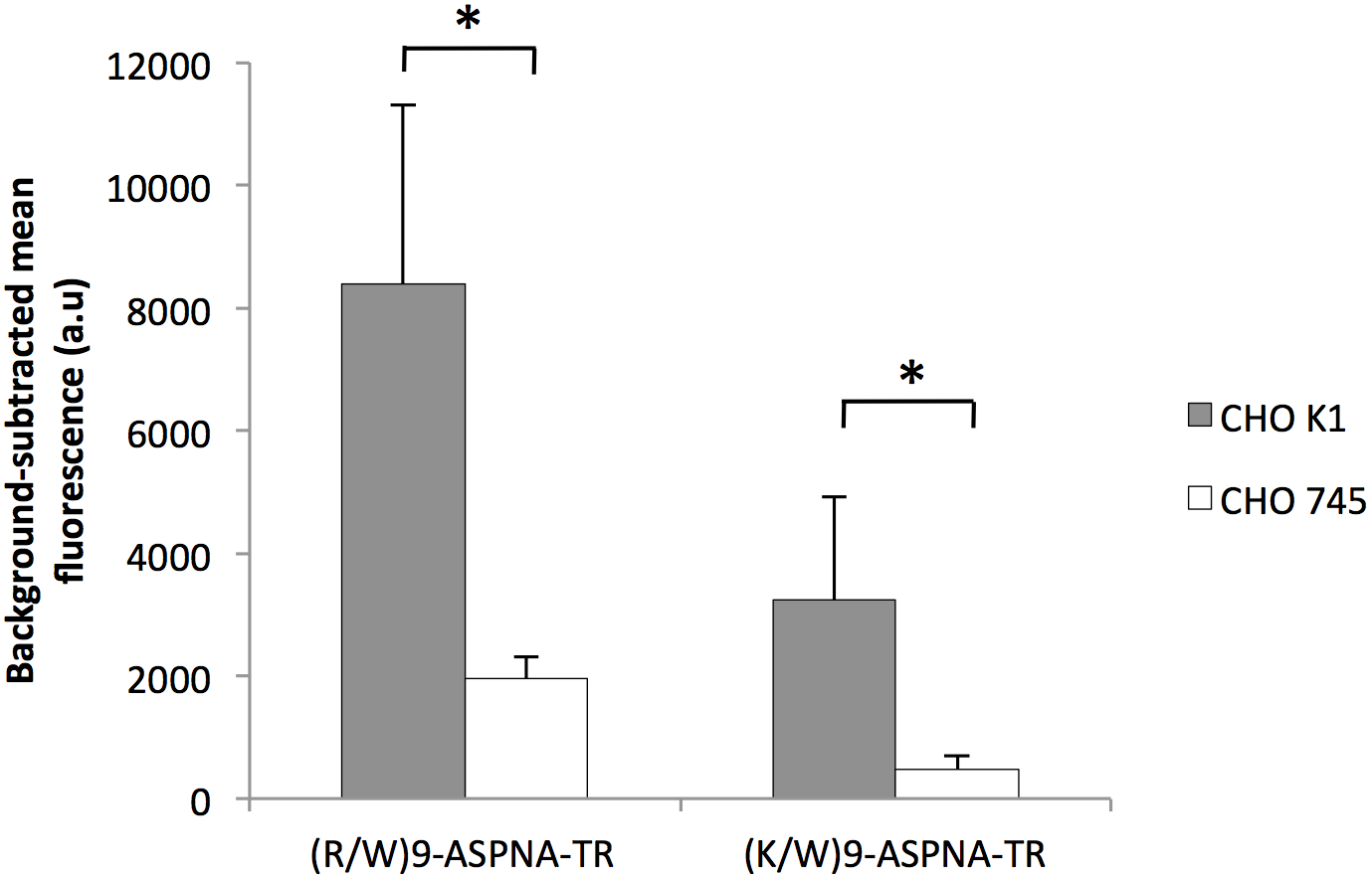
Interaction of the conjugates with cell surface glycosaminoglycans. CHO K1 and CHO 745 cells were treated with 1 µM of the indicated conjugates for 4 h. The fluorescence was quantified by FACS. The values are the means ± SD of at least three independent experiments.

To investigate whether cell surface proteoglycans affect uptake of (R/W)9-ASPNA and (K/W)9-ASPNA-TR, we evaluated uptake into parental CHO-K1 cells and mutant CHO-745 cells, in which GAG synthesis is defective due to the lack of xylosyl transferase activity. The membranes of the mutant cells have no heparin sulfate or chondroitin sulfate [28]. The uptake of both conjugates was four-fold decreased in CHO-745 cells compared to that into parental cells (**Fig. 7**). Thus, proteoglycans promote internalization of cationic peptides without distinguishing the chemical nature of their positive charges. The uptake of the conjugates once bound to the cell surface should be dependent on the formation of specific uptake-promoting interactions and may result in arginine-specific uptake.

## Discussion

In this work we showed, using a functional cell assay and fluorescence-based methods, that internalization of (R/W)9-ASPNA conjugates is energy-dependent and involves macropinocytosis and both clathrin-dependent and clathrin-independent endocytosis. Endosomal release of such conjugates for efficient antisense PNA activity was obtained by incubation of cells with chloroquine. Recently, Aubry et al. [29] showed that (R/W)9 disulfide-conjugated to a peptidic cargo can be cross-linked by cell-surface thiol proteins. In addition, they reported higher cell association and enhanced cell entry for (R/W)9 harbouring oxidized or reduced Cys residues compared to the free Cys version. In order to determine whether reduced Cys residues are important for cell uptake, we investigated the antisense activity of (R/W)9-PNA containing either a disulfide bridge or a maleimide linker after free uptake of both conjugates in PPT/HeLa cells. The disulfide bridge is used as linker to conjugate the PNA to a CPP because PNAs, like other polymers, can be easily functionalized with a thiol. Our results show that stably linked (R/W)9-PNA was slightly more active than disulfide-linked conjugate; therefore, even though there is a reduction and oxidation of thiols at the cell surface this did not interfere in the cellular association or internalization of (R/W)9-ASPNA conjugate.

The mechanism of cellular uptake of (R/W)9-ASPNA was investigated. First, we have shown that the disulfide bridge-containing conjugate was rapidly taken up by cells with levels reaching a plateau after 100 min of incubation. The observed initial rapid increase in uptake likely reflects the endosomal recycling, and the plateau stage the saturation of the endocytotic machinery. By investigating the co-localization of (R/W)9-ASPNA complexes with Alexa Fluor488-labeled dextran (marker of macropinocytosis), hTF (a CDE marker), and cholera toxin subunit B (a CIE marker), we showed that macropinocytosis, clathrin-mediated endocytosis, and caveola-mediated endocytosis all contribute to (R/W)9-ASPNA-TR uptake. This indicates that the different pinocytosis pathways are not mutually exclusive, as was previously shown for TGF-*β* internalization [30]. After internalization, the conjugates were observed in the endosomal/lysosomal compartments, as indicated by co-localization with LysoTracker green (a dye used to label lysosomes in live cells) as previously shown for other CPP-linked cargoes [22], [31]–[34]. A quantitative assessment of the contribution of each endocytic pathway to the cellular uptake of these conjugates is essential for elucidating the intracellular fate of the CPP-PNA conjugate. Entry of the conjugates through the endosomal pathway makes use of a naturally occurring cellular transport mechanism. The entrapment of the conjugate in endosomal vesicles may lead to its degradation in the lysosomes and, if the target is an mRNA, conjugates must escape from the endosomal/lysosomal compartments to elicit the PNA antisense effect. Therefore, more sophisticated cell techniques will be needed to determine how and when the conjugates escape endocytic vesicles. It has been shown that the nuclear-active fraction of PNA for splicing redirection is internalized predominantly by CME [10], [35], [36] and that endosomal trapping significantly limits antisense activity [35], [37], [38]. Torres et al. [18] showed that for miRNA targeting, CQ treatment did not significantly affect Cys-K-PNA23mer-K3 anti-miR-122 activity. These results strongly suggest that targeting of miRNAs by anti-miRs occurs within or is associated with endosomal compartments and does not require PNA release into the cytosol. In the presence of CQ, Cys-K-ASPNA-K3 and (R/W)9-ASPNA inhibited reporter gene expression by 6.5±14 and 57±5%, respectively. The significantly great antisense activity of the (R/W)9 conjugate could be due to enhanced uptake or to enhanced endosomal escape efficacy. We therefore investigated the intracellular fate of (R/W)9-ASPNA. Our microscopy images show that endosomes and lysosomes of cells transfected in the presence of CQ are larger than those in cells not treated with CQ and that the conjugate is concomitantly liberated into the cytoplasm. Most probably, CQ accumulates in PPT/HeLa cells with a cell-to-medium concentration gradient of about 100 as described for human hepatocarcinoma cells [20]. CQ unprotonated amines can absorb protons that are pumped into acidic vesicles, thus treatment with CQ results in an increased influx of Cl^−^ ions and water and endosome swelling and rupture with the release of the conjugate in the cytoplasm. The antisense activity of the (R/W)9-ASPNA conjugate measured 24 hours after CQ treatment was identical to the activity of unconjugated ASPNA in SLO-permeabilized cells, suggesting that endosomal escape is very efficient after CQ treatment. Entrapment within the endosome/lysosome compartment after endocytosis remains a limiting factor to effective use of PNA-based agents. It has been reported that at high concentrations (>3 µM) (R-Ahx-R)_4_-PNA and R_6_Pen-PNA conjugates induce splicing redirection in PPT/HeLa cells in the absence of endosomolytic agents; however, the low efficacy and the toxicity due to the high concentration of arginine residues limit the use of these conjugates [35]. Gait’s group attempted to improve the efficacy of (R-Ahx-R)_4_-PNA conjugates by synthesizing branched, arginine-rich dendrimer conjugates. However, 8-Arg branched peptide-PNA conjugates showed poor activity compared to the linear conjugate [10]. Nielsen’s team showed that conjugation of fatty acid to the cationic peptide increases the biological effect of the corresponding PNA conjugates up to two orders of magnitude [39]. The effect increased with increasing length of the fatty acid chain but in parallel resulted in increased cellular toxicity.

We then investigated the role of the arginine residues of (R/W)9 in the cell uptake and biological activity of the conjugates. The cell uptake of the (K/W)9-ASPNA variant, in which all arginine where mutated in lysines, was decreased six fold compared to (R/W)9-ASPNA in PPT/HeLa cells and in four other cell lines, indicating that the arginine residues confer a cell uptake advantage to (R/W)9 conjugates. It has been reported also that a penetratin variant in which all lysine residues were substituted with arginines (PenArg) is internalized more efficiently than penetratin itself [40], [41]. We show that the uptake advantage of (R/W)9-ASPNA conjugate is correlated with reduction in cytoplasmic mRNA target expression. Moreover, the strong decrease in (R/W)9-ASPNA and (K/W)9-ASPNA cell uptake into CHO-745 cells, in which proteoglycan synthesis is defective, shows that glycosaminoglycans are necessary for cell surface binding and internalization of both conjugates. It has been shown that penetratin and PenArg binding to sulfated sugars is stabilized by hydrophobic interactions and result in clustering, whereas PenLys only interacts electrostatically with sugars [41]. It is noteworthy that the free uptake studies of CPPs indicate that tryptophan content and backbone spacing within basic peptide also affect uptake efficiency [42], [43].

Our study shows that the nonapeptide containing six arginines and three tryptophans is a very effective carrier for PNA targeting cytoplasmic mRNA. PNA-CPP mediated gene targeting strategy has been used recently *in* *vivo*. Rogers et al. [44] reported that triplex-forming PNAs covalently linked to the CPP Antp modify a target gene in hematopoietic progenitor cells of mice following systemic administration via intraperitoneal injection. We have shown that a PNA targeted to the coding region of *IGF-1R* mRNA inhibits translation elongation and cell transformation when introduced by electroporation to human prostate cancer cells grown in culture [45]. IGF-1R is overexpressed in many tumors and is an important target in cancer therapy. We expect that the data reported here on (R/W)9-ASPNA cellular activity will guide design of new therapeutic candidates. The use of CQ *in* *vivo* is not a limiting step since a relevant adjuvant effect was observed in patients with glioblastoma multiforme when CQ was added to the treatment regimen [46]. In addition, it was recently shown that short interfering DNA (Dbait) covalently attached to cholesterol exerts an antitumoral effect on xenografted melanoma when mice were pre-treated with CQ [24].

## Supporting Information

Figure S1 **Cells were reversibly permeabilized using SLO in the presence of 1** µ**M (final concentration) of unconjugated or (R/W)9-conjugated PNAs.** Luciferase activity was measured 24 h later and is expressed as luminescence/mg of protein (arbitrary units, a.u.). Percentages are relative to activity in untreated cells. The values represent the mean ± SD of at least four independent experiments. ASPNA, antisense PNA; SCRPNA, scramble PNA; S-S, disulfide linker; mal, maleimide linker.
(TIFF)

Figure S2 **PPT/HeLa cells were incubated with unconjugated ASPNA or (R/W)9-conjugated ASPNA at 1.5** µ**M concentration with (grey bars) or without (white bars) 150** µ**M chloroquine (CQ) for 4** **h in serum-free or 7% FCS DMEM medium.** Cell viability was analyzed 24 h after addition of doxycycline using the MTS cell proliferation assay, and values were normalized to the average value of the untreated sample. Values are the mean ± SD of at least three independent experiments.
(TIFF)

Figure S3 **Relative luciferase activity in PPT/HeLa cells incubated with unconjugated or (R/W)9-conjugated ASPNA at 1.5**µ**M with (grey bars) or without (white bars) 150**µ**M chloroquine (CQ) for 4** **h in 7% FCS DMEM medium.** Luciferase activity was measured 24 h after addition of doxycycline and expressed as luminescence/mg of protein. Percentages are relative to luciferase activity in untreated cells.
(TIFF)

Figure S4 **U2OS (dark grey bars), MCF-7 (white bars), DU-145 (black bars), and Jurkat cells (light grey bars) were incubated with 1** µ**M (R/W)9-ASPNA or 1** µ**M (K/W)9-ASPNA for 4** **h and the fluorescence was quantified by FACS.** Values represent the means ± SD of at least three independent experiments.
(TIFF)

Figure S5 **PPT/HeLa cells permeabilized with streptolysin-** ***O*** **were transfected with 1** µ**M unconjugated ASPNA or ASPNA conjugated to (R/W)9 or (K/W)9.** Luciferase activity was measured 24 h later and is expressed as luminescence/mg of protein. Percentages are relative to untreated cells. Values represent the means ± SD of at least three independent experiments.
(TIFF)
