# Supplementary figures and images for: Delivery of Antisense Peptide Nucleic Acids to Cells by Conjugation with Small Arginine-Rich Cell-Penetrating Peptide (R/W)9
Source: PLoS One. 2014 Aug 15;9(8):e104999. doi: 10.1371/journal.pone.0104999 (PMC4134252; doi:10.1371/journal.pone.0104999)

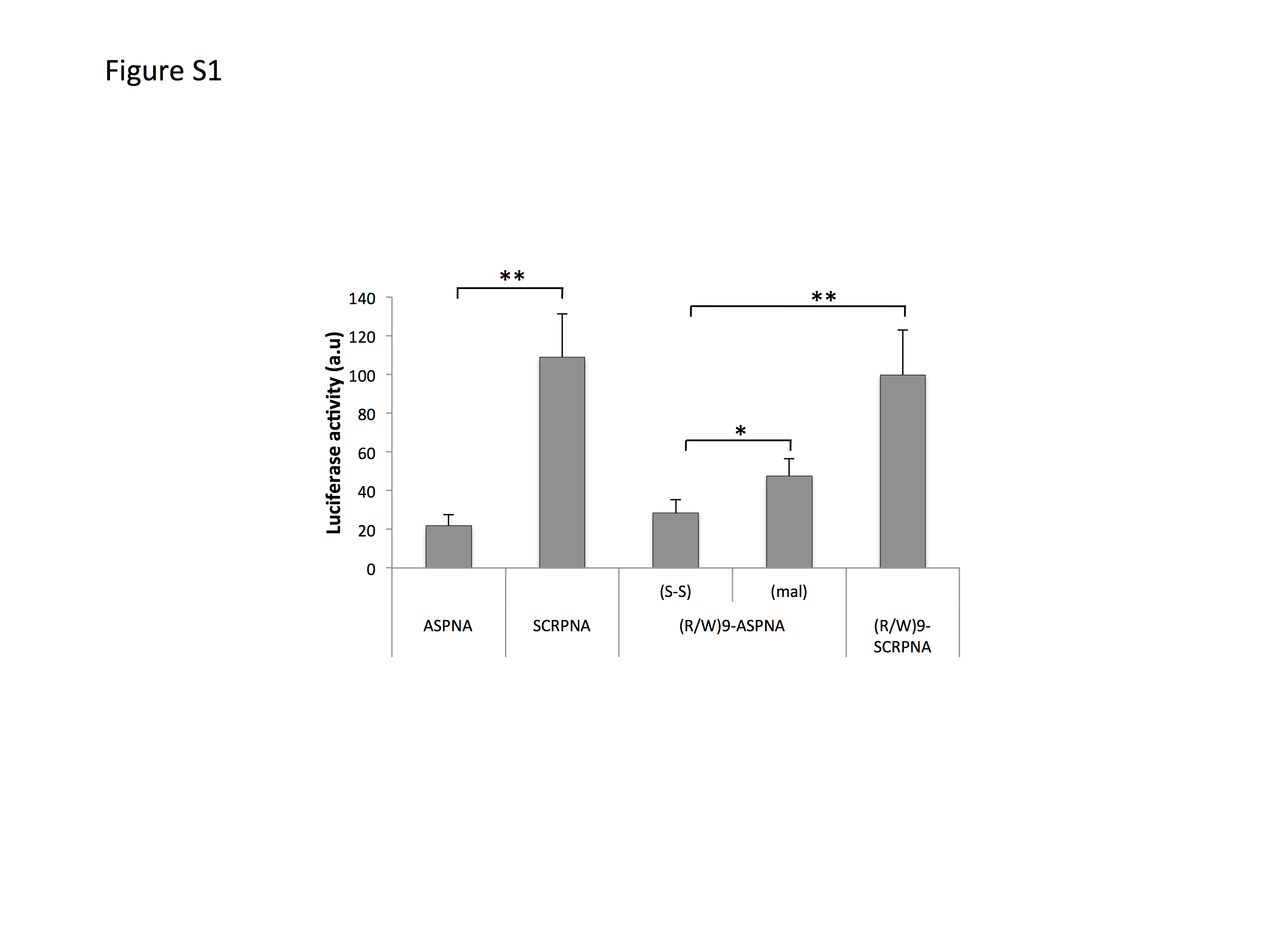

Supplement: Figure S1 — Cells were reversibly permeabilized using SLO in the presence of 1 µM (final concentration) of unconjugated or (R/W)9-conjugated PNAs. Luciferase activity was measured 24 h later and is expressed as luminescence/mg of protein (arbitrary units, a.u.). Percentages are relative to activity in untreated cells. The values represent the mean ± SD of at least four independent experiments. ASPNA, antisense PNA; SCRPNA, scramble PNA; S-S, disulfide linker; mal, maleimide linker. (TIFF) [file pone.0104999.s001.tiff]

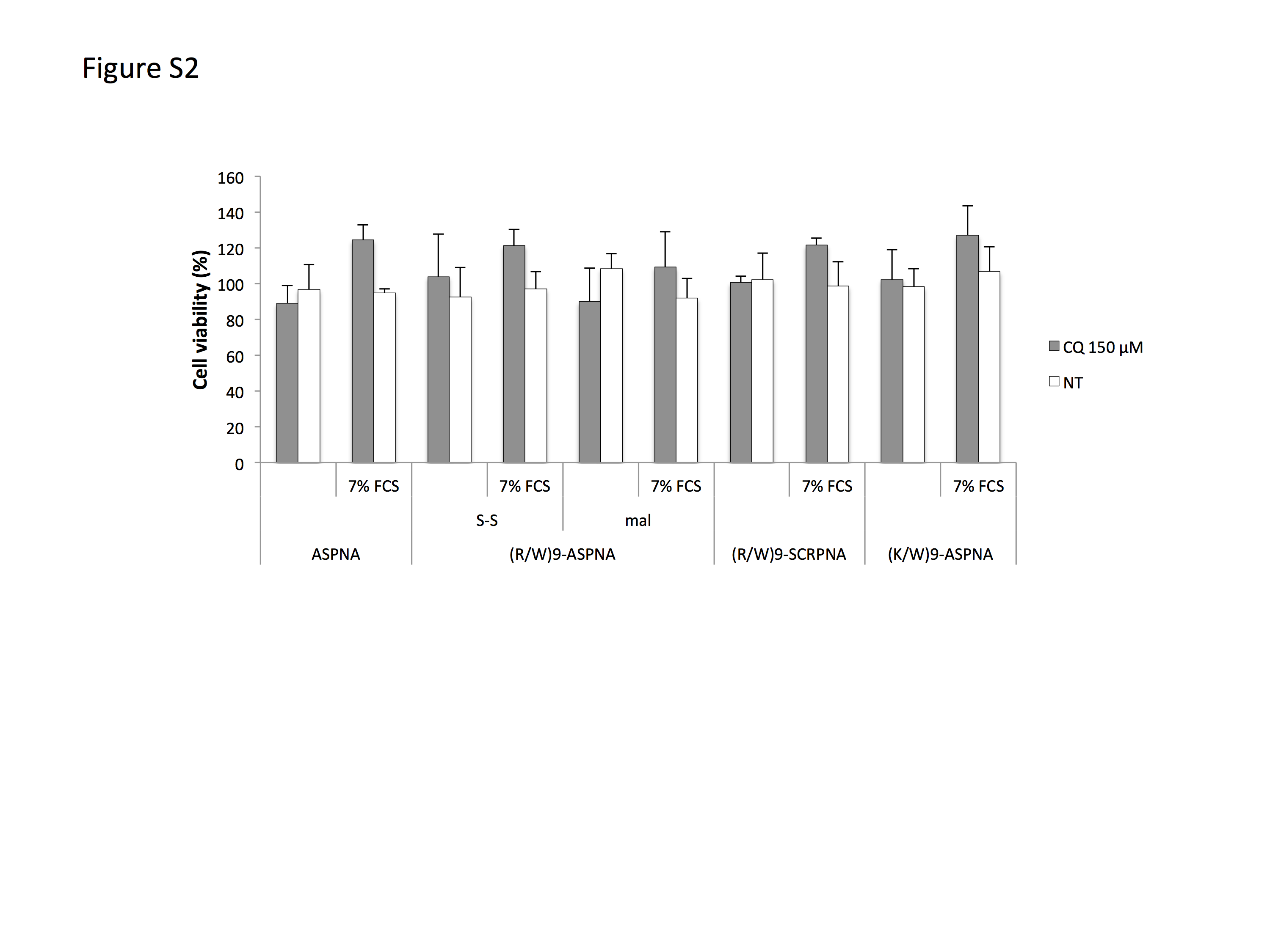

Supplement: Figure S2 — PPT/HeLa cells were incubated with unconjugated ASPNA or (R/W)9-conjugated ASPNA at 1.5 µM concentration with (grey bars) or without (white bars) 150 µM chloroquine (CQ) for 4 h in serum-free or 7% FCS DMEM medium. Cell viability was analyzed 24 h after addition of doxycycline using the MTS cell proliferation assay, and values were normalized to the average value of the untreated sample. Values are the mean ± SD of at least three independent experiments. (TIFF) [file pone.0104999.s002.tiff]

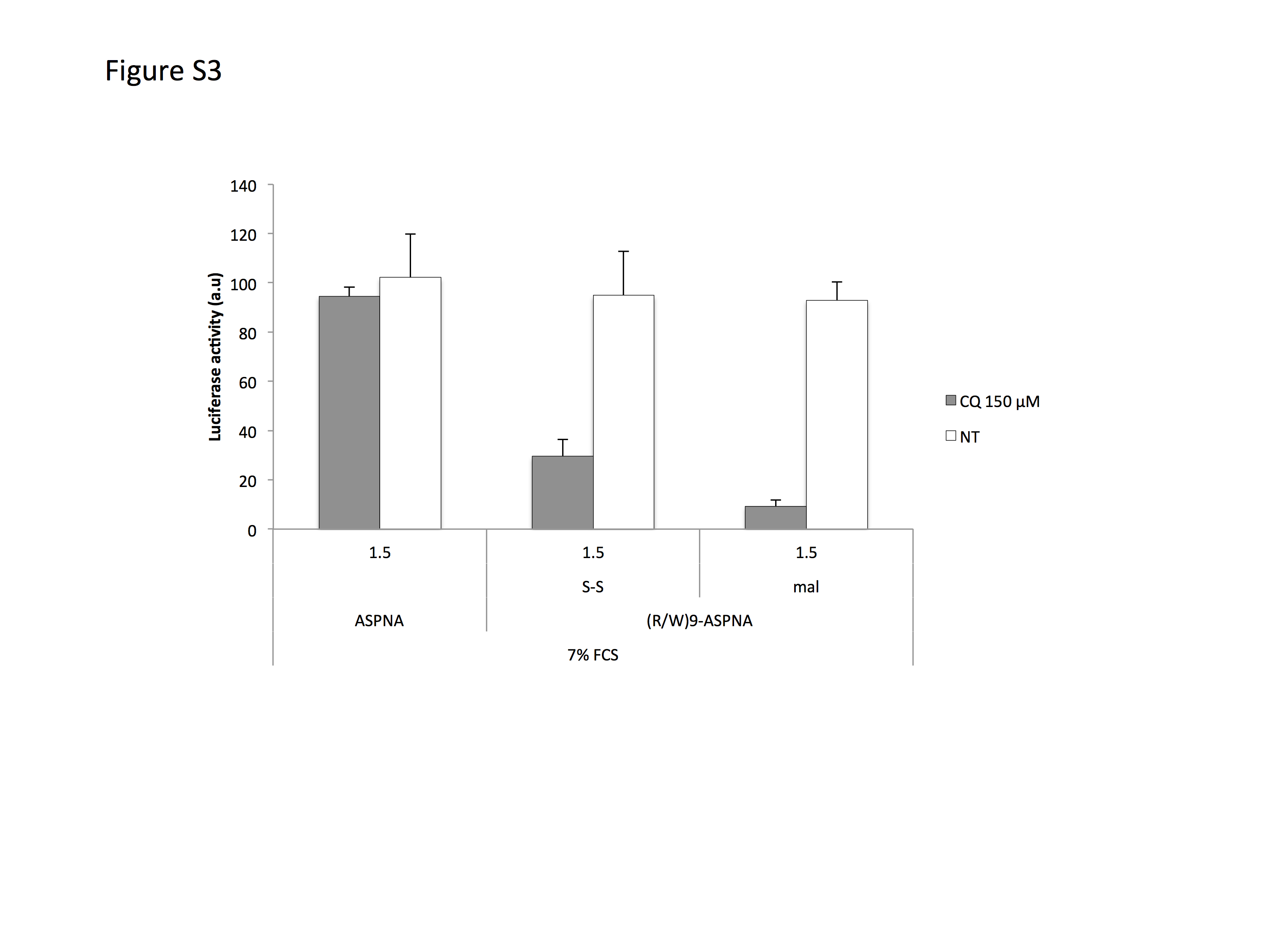

Supplement: Figure S3 — Relative luciferase activity in PPT/HeLa cells incubated with unconjugated or (R/W)9-conjugated ASPNA at 1.5µM with (grey bars) or without (white bars) 150µM chloroquine (CQ) for 4 h in 7% FCS DMEM medium. Luciferase activity was measured 24 h after addition of doxycycline and expressed as luminescence/mg of protein. Percentages are relative to luciferase activity in untreated cells. (TIFF) [file pone.0104999.s003.tiff]

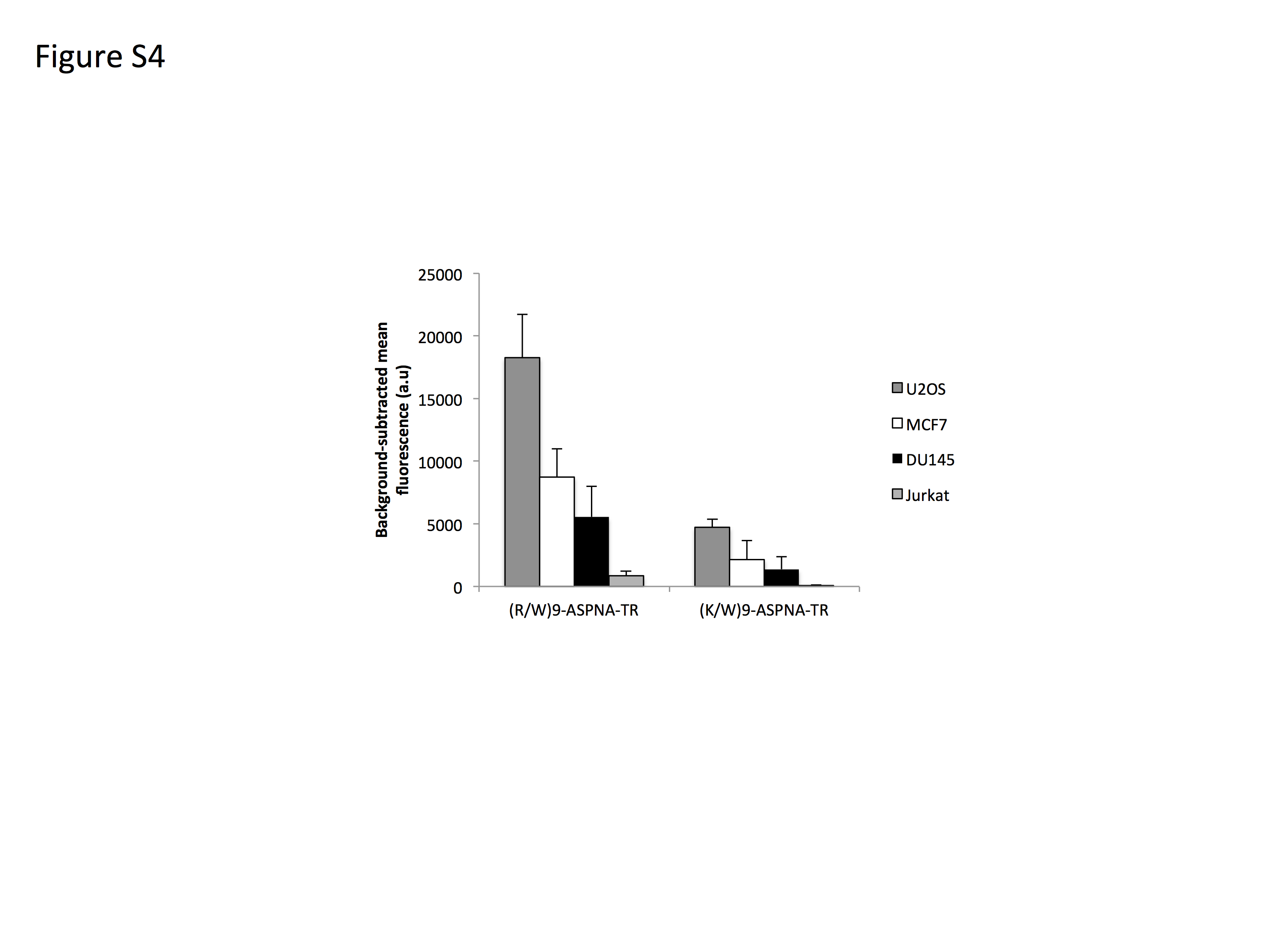

Supplement: Figure S4 — U2OS (dark grey bars), MCF-7 (white bars), DU-145 (black bars), and Jurkat cells (light grey bars) were incubated with 1 µM (R/W)9-ASPNA or 1 µM (K/W)9-ASPNA for 4 h and the fluorescence was quantified by FACS. Values represent the means ± SD of at least three independent experiments. (TIFF) [file pone.0104999.s004.tiff]

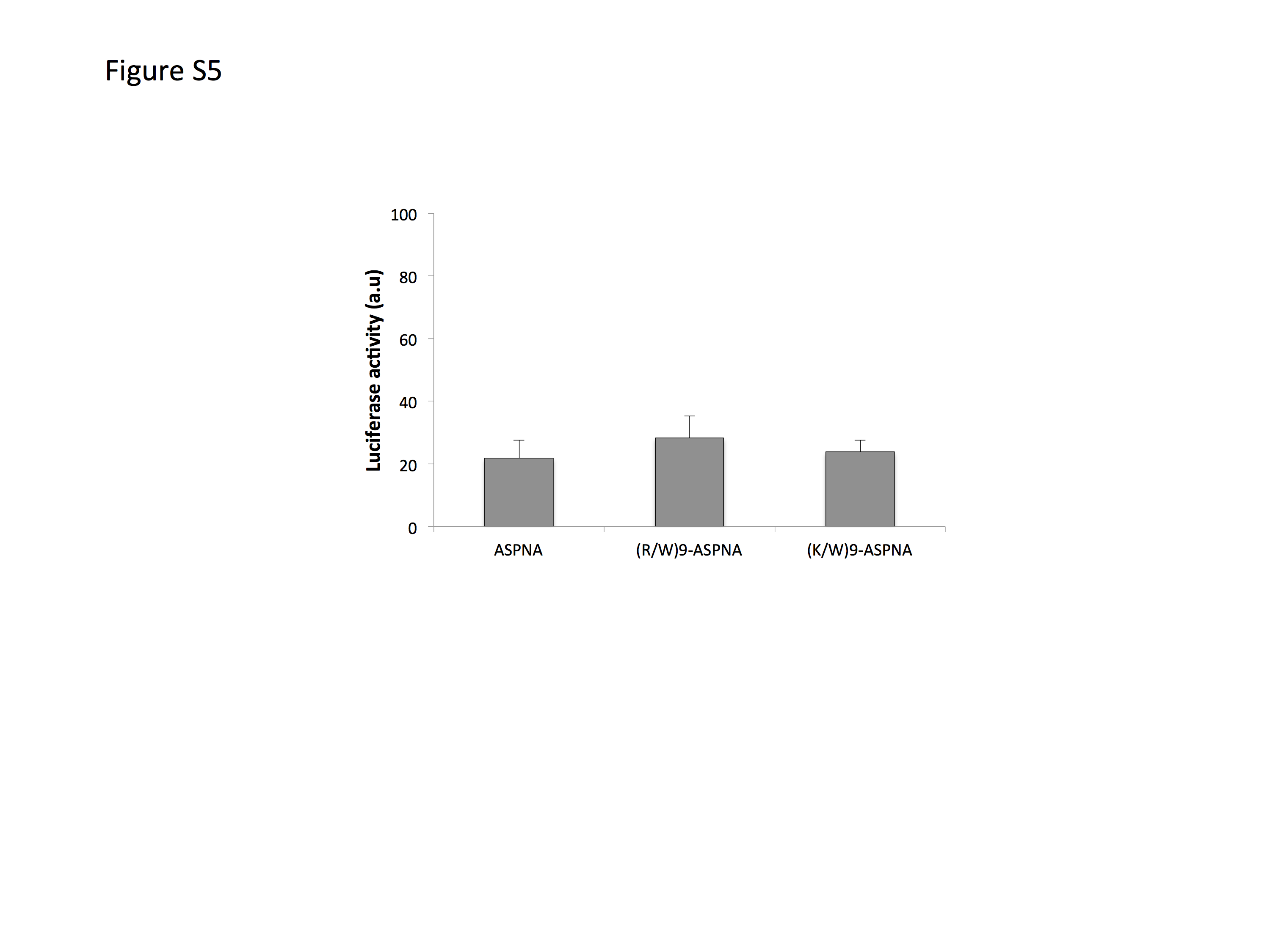

Supplement: Figure S5 — PPT/HeLa cells permeabilized with streptolysin- O were transfected with 1 µM unconjugated ASPNA or ASPNA conjugated to (R/W)9 or (K/W)9. Luciferase activity was measured 24 h later and is expressed as luminescence/mg of protein. Percentages are relative to untreated cells. Values represent the means ± SD of at least three independent experiments. (TIFF) [file pone.0104999.s005.tiff]
